# Somatostatin contributes to long-term potentiation at excitatory synapses onto hippocampal somatostatinergic interneurons

**DOI:** 10.1186/s13041-021-00830-6

**Published:** 2021-08-24

**Authors:** Anne-Sophie Racine, François-Xavier Michon, Isabel Laplante, Jean-Claude Lacaille

**Affiliations:** Centre for Interdisciplinary Research on Brain and Learning, Research Group on the Central Nervous System, Department of Neurosciences, Université de Montréal, P.O. Box 6128, Station Downtown, Montreal, QC H3C 3J7 Canada

**Keywords:** GABA interneurons, Somatotropin-release inhibitory factor—SRIF, Whole cell recordings, Cysteamine, SST_1-5_ receptors, Disinhibition, GABA_A_ inhibition, Hebbian LTP

## Abstract

Somatostatin-expressing interneurons (SOM-INs) are a major subpopulation of GABAergic cells in CA1 hippocampus that receive excitation from pyramidal cells (PCs), and, in turn, provide feedback inhibition onto PC dendrites. Excitatory synapses onto SOM-INs show a Hebbian long-term potentiation (LTP) mediated by type 1a metabotropic glutamate receptors (mGluR1a) that is implicated in hippocampus-dependent learning. The neuropeptide somatostatin (SST) is also critical for hippocampal long-term synaptic plasticity, as well as learning and memory. SST effects on hippocampal PCs are well documented, but its actions on inhibitory interneurons remain largely undetermined. In the present work, we investigate the involvement of SST in long-term potentiation of CA1 SOM-IN excitatory synapses using pharmacological approaches targeting the somatostatinergic system and whole cell recordings in slices from transgenic mice expressing eYFP in SOM-INs. We report that application of exogenous SST_14_ induces long-term potentiation of excitatory postsynaptic potentials in SOM-INs via somatostatin type 1–5 receptors (SST_1-5_Rs) but does not affect synapses of PC or parvalbumin-expressing interneurons. Hebbian LTP in SOM-INs was prevented by inhibition of SSTRs and by depletion of SST by cysteamine treatment, suggesting a critical role of endogenous SST in LTP. LTP of SOM-IN excitatory synapses induced by SST_14_ was independent of NMDAR and mGluR1a, activity-dependent, and prevented by blocking GABA_A_ receptor function. Our results indicate that endogenous SST may contribute to Hebbian LTP at excitatory synapses of SOM-INs by controlling GABA_A_ inhibition, uncovering a novel role for SST in regulating long-term synaptic plasticity in somatostatinergic cells that may be important for hippocampus-dependent memory processes.

**Supplementary Information:**

The online version contains supplementary material available at 10.1186/s13041-021-00830-6.

## Introduction

Hippocampal GABAergic interneurons are highly heterogenous with different types distinguished according to their morphology, connectivity, physiologic characteristics, and molecular makers [1]. Somatostatin-expressing interneurons (SOM-INs) are a major subpopulation of GABAergic cells [2]. Characteristically, CA1 SOM-INs receive a major excitation from local pyramidal cells (PCs), and, in turn, provide feedback inhibition onto dendrites of PCs [3]. SOM-INs are comprised of distinct subtypes, including the Oriens-Lacunosum/Moleculare (O-LM) cells, bistratified cells, and also projection cells with additional subicular, retro-hippocampal or septal projections [3, 4]. SOM-INs regulate PC synaptic integration [5], action potential rate and burst firing [6] as well as synaptic plasticity [7–9], and play a critical role in contextual fear learning [10, 11].

A notable feature of CA1 SOM-INs is the long-term plasticity occurring at their excitatory synapses. These synapses show a Hebbian long-term potentiation (LTP) mediated by type 1a metabotropic glutamate receptors (mGluR1a) [8, 12, 13]. Excitatory synapses onto parvalbumin-expressing interneurons (PV-INs) do not display this form of long-term plasticity [8]. In addition, SOM-INs excitatory synapses show a late form of mGluR1a-dependent LTP, that can last from a few to 24 h and involves mammalian target of rapamycin complex 1 (mTORC1) mediated translation [9, 14, 15]. Interestingly, cell-specific conditional down-regulation of mTORC1 in SOM-INs impairs late mGluR1a-dependent LTP, as well as contextual fear and spatial memory consolidation [9]. Conversely, conditional up-regulation of mTORC1 activity in SOM-INs facilitates late mGluR1a-dependent LTP, as well as hippocampal-dependent memory [9]. Contextual fear learning induces mGluR1a- and mTORC1-dependent LTP at SOM-IN excitatory synapses, suggesting a critical implication of SOM-IN long-term synaptic plasticity in hippocampal learning and memory [9]. More recently, cell-specific conditional knock-in of the non-phosphorylatable translation initiation factor eIF2α (eIF2α^S51A^) in SOM interneurons was found to upregulate general mRNA translation in these cells and be sufficient to gate CA1 network plasticity and increase long-term contextual fear memory, further supporting a critical role of SOM-INs in hippocampal long-term memory consolidation [11].

Somatostatin (SST; also known as somatotropin-release inhibitory factor, SRIF) is a peptide expressed in central nervous system. It was first discovered in the hypothalamus where it exerts an inhibitory action on growth hormone [16]. SST is implicated in multiple brain functions like olfaction, vision, cognition and locomotion, as well as in pathologies such as Alzheimer’s disease, schizophrenia and major chronic depression [17]. SST acts via five metabotropic receptors (SST_1_R to SST_5_R) that are coupled to G proteins and target many effectors [18]. SSTRs have a wide distribution in brain with overlapping regional localization of receptor types [19]. In the hippocampus, mRNA for all five SSTRs is present, although expression is weaker for SST_5_R [20, 21]. Subcellular localization of SSTRs is highly specific to the receptor type. SST_1_R is targeted pre-synaptically to axons, while SST_2,4,5_R are mostly distributed post-synaptically to neuronal somata and dendrites, and SST_3_R appears excluded from classic pre- and post-synaptic sites [19]. Consistent with the subcellular localization of its receptors, SST modulates neuronal activity via both pre- and post-synaptic mechanisms [17, 18]. In the hippocampus, exogenous SST_14_ hyperpolarizes PCs by activation of two distinct K^+^ currents (M-current and voltage-insensitive leak current) [22–24]. SST_14_ also induces a presynaptic inhibition of excitatory synaptic transmission in hippocampal PCs [25]. The presynaptic inhibition may involve a G-protein mediated inhibition of N-type voltage-gated Ca^2+^ channels [26, 27] and activation of presynaptic K^+^ channels [25]. Although SST_14_ presynaptic inhibition of hippocampal excitatory synaptic transmission is well documented, presynaptic inhibition of GABAergic inhibitory synaptic transmission has also been reported [28] as in other brain regions [29].

SST is critical for hippocampal long-term synaptic plasticity, as well as learning and memory. Depletion of SST by cysteamine treatment, or knock-out of the SST gene in transgenic mice, impairs contextual fear memory but not auditory fear learning [30]. The memory impairment is associated with a decrease in LTP in CA1 PCs [30]. Interestingly, blocking LTP at excitatory synapses of SOM-INs was found to impair contextual fear memory and facilitation of LTP in PCs by SOM-INs [9]. The analogous effects of manipulating SST or SOM-IN synaptic plasticity on contextual fear memory and PC synaptic plasticity, suggest a possible link between SST and long-term plasticity at SOM-IN excitatory synapses.

Here, we investigate the involvement of SST in long-term potentiation of CA1 SOM-IN excitatory synapses using pharmacological approaches targeting the somatostatinergic system and whole cell recordings in slices from transgenic mice expressing eYFP in SOM-INs. We report that application of exogenous SST_14_ induces long-term potentiation of excitatory postsynaptic potentials (EPSPs) of SOM-INs via SST_1-5_Rs, but not of PC and PV-IN synapses. Also, Hebbian LTP in SOM-INs was prevented by inhibition of SSTRs and depletion of SST by cysteamine treatment, suggesting a critical role of endogenous SST in LTP. LTP of SOM-IN synapses induced by SST_14_ was independent of NMDAR and mGluR1a, activity-dependent, and prevented by blocking GABA_A_ receptor function. Our results indicate that endogenous SST may contribute to Hebbian LTP at excitatory synapses of SOM-INs by controlling GABA_A_ inhibition, uncovering a novel role for SST in regulating long-term synaptic plasticity in somatostatinergic cells that may be important for hippocampus-dependent memory processes.

## Results

**Fig. 1:**
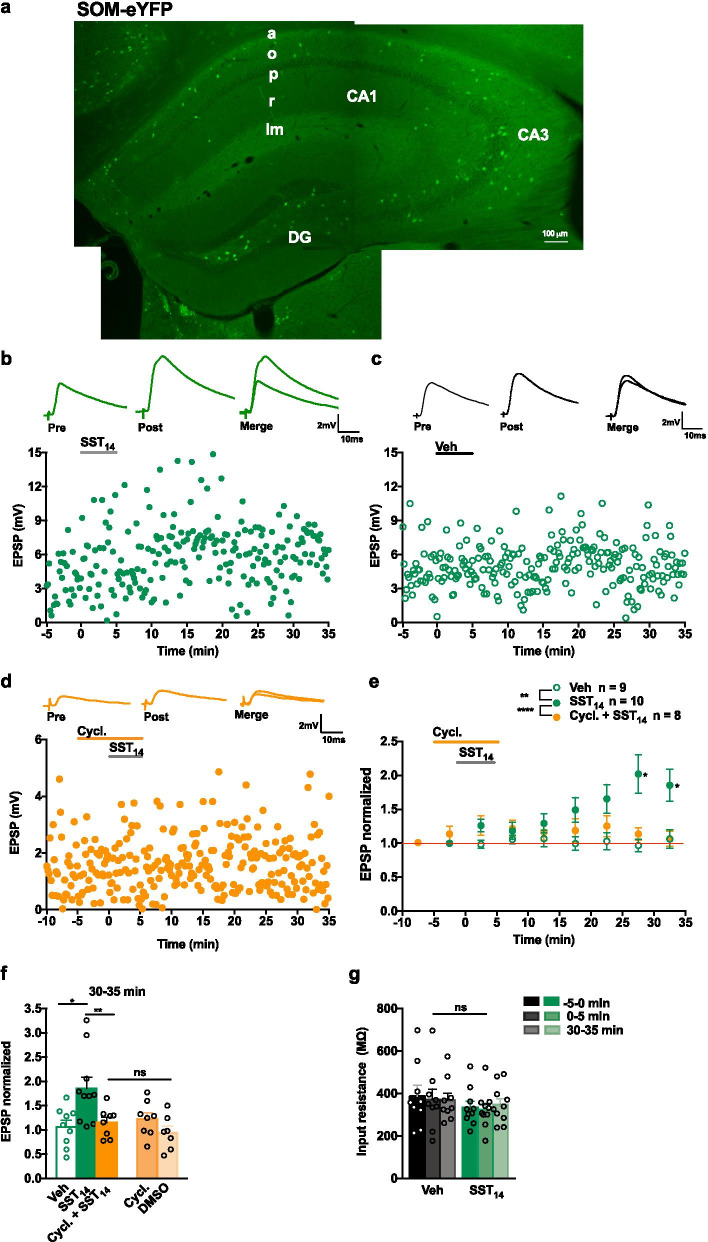
SST_14_ induces a long-term potentiation of EPSPs in SOM-INs via SSTRs. **a** Fluorescence images showing eYFP expression in SOM-INs in hippocampus of SOM-eYFP mouse. **b**–**d** Current clamp recording of EPSPs (top) and time plots of EPSP amplitude from representative cells receiving 5 µM SST_14_ (**b**), vehicle (**c**) or SST_14_ in the presence of 1 µM of the SST_1-5_R antagonist cyclosomatostatin (cycl. + SST_14_) (**d**). EPSPs are average for − 5 to 0 min baseline (pre) and 30 to 35 min after application (post). **e** Summary time plots of EPSPs (normalized to baseline), showing potentiation of EPSPs after SST_14_ application (filled green circle), prevented by co-application of the SST_1-5_R antagonist cyclosomatostatin (filled orange square), and lack of effect of vehicle application (open green circle). For SST_14_ group, n = 10 cells and 9 mice, rmANOVA, Dunnett’s multiple comparisons (25–30 min *p* = 0.025, 30–35 min *p* = 0.023). For vehicle group, n = 9 cells and 6 mice, rmANOVA *p* = 0.864. For cyclostomatostatin + SST_14_ group, n = 8 cells and 5 mice, rmANOVA *p* = 0.382. **f** Summary bar graph of EPSP amplitude at 30–35 min after application, showing long-term potentiation after SST_14_, and no effect after either SST_14_ with SST_1-5_R antagonist, vehicle, cyclosomatostatin alone, or DMSO (vehicle for cyclosomatostatin) (Veh *vs* SST_14_ group, two-way mixed ANOVA, univariate analysis at 30–35 min p = 0.010; SST_14_ *vs* Cycl. + SST_14_ group, two-way mixed ANOVA, univariate analysis at 30–35 min *p* = 0.002; Cycl. + SST_14_ *vs* Cycl. and *vs* DMSO group, two-way mixed ANOVA *p* = 0.602). **g** Summary bar graph showing no change in cell input resistance before (− 5–0 min), during (0–5 min) and after (30–35 min) vehicle or SST_14_ application. Two-way mixed ANOVA, *p* = 0.554. *p < 0.05; **p < 0.01; *ns* not significant

The excitatory synapses onto CA1 SOM-INs show long-term plasticity [8, 9]. Here we investigate if the peptide SST_14_, that is expressed specifically in SOM-INs, is involved in long-term plasticity of their excitatory synapses, using whole cell recordings in acute slices from SOM-eYFP mice (*Sst*^ires−Cre^;*Rosa26*^lsl−EYFP^) that express eYFP in SOM-INs (Fig. 1a). Statistical results are summarized in the supplemental statistical table (Additional file 1).

### *SST*_*14*_* induces LTP *via* SSTRs*

We examined with current clamp recordings the effects of application of exogenous SST_14_ on EPSPs evoked in eYFP-expressing SOM-INs in CA1 *stratum oriens*. Bath application of 5 µM SST_14_ for 5 min induced a gradual slow onset potentiation of EPSP amplitude that developed over 10–35 min after SST_14_ application (201.9 ± 28.1% of control at 25–30 min; 185.4 ± 23.4% of control at 30–35 min; Fig. 1b, e, f). Similar vehicle application did not induce change in EPSPs over the same time period, ruling out non-specific effects due to recording conditions (96.8 ± 9.4% of control at 25–30 min; 106.1 ± 13.4% of control at 30–35 min; Fig. 1c, e, f). These results indicate that exogenous SST_14_ induces long-term potentiation of EPSPs in SOM-INs.

We verified that the effects of SST_14_ were mediated by SSTRs using the non-selective SST_1-5_R antagonist cyclosomatostatin [31]. Co-application of 1 µM cyclosomatostatin with SST_14_ blocked the long-lasting increase in EPSP amplitude induced by SST_14_ (113.4 ± 9.4% of control at 25–30 min; 106.8 ± 11.4% of control at 30–35 min; Fig. 1d–f). These results indicate that the potentiation induced by exogenous SST_14_ is mediated by SST_1-5_Rs, thus ruling out non-specific drug effects of SST_14_. Interestingly, bath application of cyclosomatostatin alone, or its vehicle DMSO, did not affect EPSPs (122.7 ± 12.8% and 94.0 ± 13.4% of control at 30–35 min, respectively; Fig. 1f), suggesting the absence of endogenous activation of SST_1-5_Rs and modulation of EPSPs during low frequency stimulation alone.

Bath application of SST_14_ affected EPSPs but had no effect on cell input resistance in the same cells. Cell input resistance was unchanged during and after SST_14_ or vehicle application (96.1 ± 13.3% of control during and 95.3 ± 9.1% of control at 30–35 min after SST_14_; 97.1 ± 8.5% of control during and 103.8 ± 8.0% of control at 30–35 min after vehicle; Fig. 1g). Thus, SST_14_ effects on EPSPs may not involve postsynaptic changes in cell input resistance.

### *SST*_*1-5*_*R antagonist or cysteamine treatment prevent Hebbian LTP*

Excitatory synapses onto CA1 SOM-INs show a mGluR1a-dependent Hebbian LTP [8, 12]. Since SST_14_ induces LTP of EPSPs in SOM-INs via SSTRs, next we examined if endogenous SST could be involved in Hebbian LTP.

**Fig. 2:**
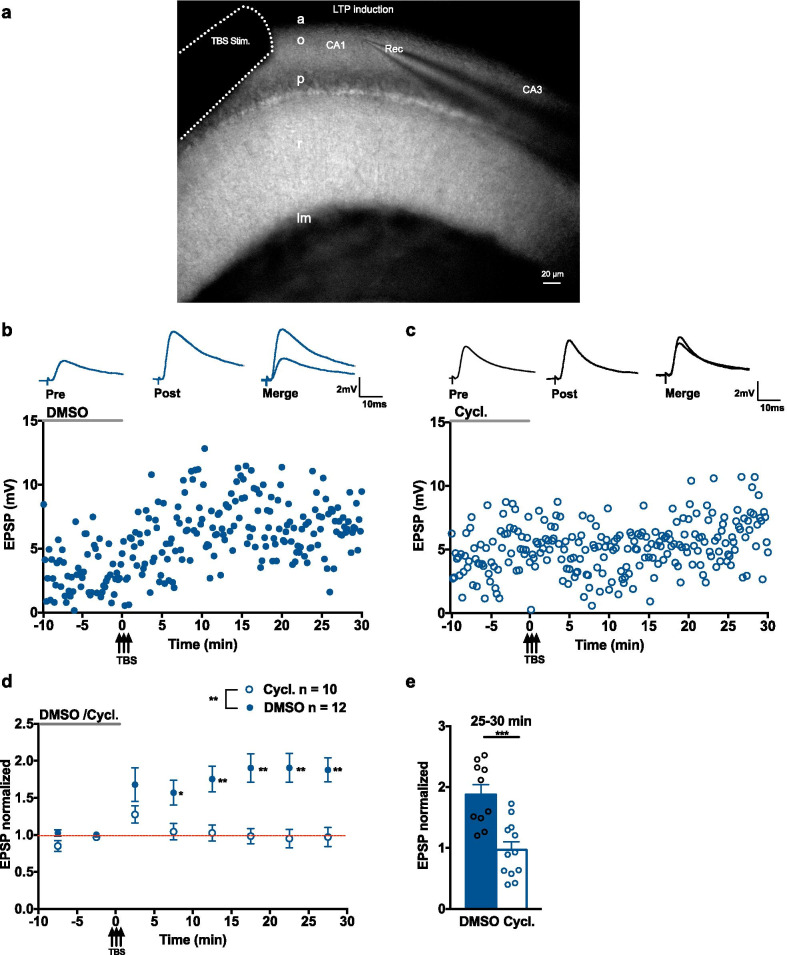
SST_1-5_R antagonist cyclosomatostatin prevents Hebbian LTP. **a** Image showing microelectrodes configuration for stimulation and recording during TBS-induced LTP experiments. **b**, **c** Current clamp recording of EPSPs (top) and time plots of EPSP amplitude from representative cells receiving TBS in the presence of DMSO (0.01%) (**b**) or the SST_1-5_R antagonist cyclosomatostatin (1 µM) (**c**). **d** Summary time plots of EPSPs (normalized to baseline), showing that TBS in the presence of DMSO induces LTP of EPSPs (filled blue circle), but TBS in the presence of cyclosomatostatin does not (open blue circle). For DMSO group, n = 10 cells and 6 mice, rmANOVA, Dunnett’s multiple comparisons (5–10 min *p* = 0.040, 10–15 min *p* = 0.009, 15–20 min *p* = 0.006, 20–25 min *p* = 0.006, 25–30 min *p* = 0.002). For cyclosomatostatin group, n = 12 cells and 6 mice, rmANOVA, *p* = 0.124. **e** Summary bar graph of EPSP amplitude at 25–30 min after TBS showing LTP in DMSO but not in cyclosomatostatin. Two-way mixed ANOVA with univariate analysis of variance, 25–30 min *p* = 0.0003. *p < 0.05; **p < 0.01; ***p < 0.001; *ns* not significant

We used whole cell current clamp recordings to monitor Hebbian LTP elicited in SOM-INs by theta burst stimulation (TBS), and bath application of the SST_1-5_R antagonist cyclosomatostatin to test for a possible role of SST (Fig. 2). Cyclosomatostatin or its vehicle (DMSO) were applied 10 min prior to and during TBS, and then washed out (Fig. 2b–d). In the presence of DMSO, TBS induced a slow onset LTP of EPSP amplitude (157.0 ± 16.7% of control at 5–10 min; 175.3 ± 17.2% of control at 10–15 min; 190.3 ± 19.2% of control at 15–20 min; 190.4 ± 19.4% of control at 20–25 min; 187.7 ± 16.2% of control at 25–30 min; Fig. 2d, e). However, in the presence of 1 µM cyclosomatostatin, a concentration that prevents SST_14_ potentiation of EPSPs, TBS failed to induce LTP of EPSP amplitude (97.1 ± 13.1% of control at 25–30 min; Fig. 2d, e). These results suggest that endogenous SST may be released and activate SSTRs in Hebbian LTP induced by TBS in SOM-INs.

**Fig. 3:**
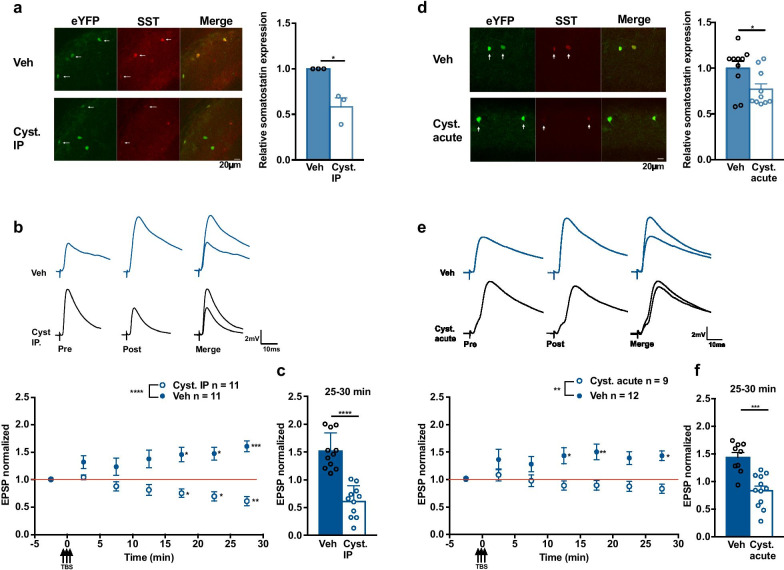
Cysteamine treatment lowers SST levels and prevents Hebbian LTP. **a** Representative images (left) and summary bar graph (right) showing reduction of SST immunofluorescence in eYFP-expressing SOM-INs after cysteamine injection (vehicle n = 3 mice, cysteamine n = 3 mice, unpaired *t*-test *p* = 0.047). **b** EPSPs from representative cells (top) and summary time plots of EPSPs (bottom), showing LTP induced by TBS in vehicle injected mice (filled blue square), and absence of LTP (but EPSP depression) in cysteamine injected mice (open blue circle). For vehicle group, n = 11 cells and 8 mice, rmANOVA, Dunnett’s multiple comparisons (15–20 min *p* = 0.028, 20–25 min *p* = 0.016, 25–30 min *p* = 0.0007). For cysteamine group, n = 11 cells and 6 mice, rmANOVA, Dunnett’s multiple comparisons (15–20 min *p* = 0.040, 20–25 min *p* = 0.026, 25–30 min *p* = 0.005). **c** Summary bar graph, showing absence of LTP at 25–30 min after TBS in cysteamine injected mice (two-way mixed ANOVA with univariate analysis of variance, *p* < 0.0001). **d**–**f** Similar data presentation showing effects of incubation for one hour of slices with cysteamine. (**d**) Cysteamine treatment reduces SST immunofluorescence in SOM-INs (vehicle n = 4 mice, cysteamine n = 4 mice, unpaired t-test p = 0.029). (**e**) Cysteamine treatment prevents TBS induction of LTP. For vehicle group, n = 9 cells and 7 mice, rmANOVA, Dunnett’s multiple comparisons (10–15 min *p* = 0.027, 15–20 min *p* = 0.008, 25–30 min *p* = 0.027). For cysteamine group, n = 12 cells and 7 mice, rmANOVA *p* = 0.097. (**f**) Summary bar graph showing absence of LTP after cysteamine treatment (two-way mixed ANOVA with univariate analysis of variance, 25–30 min p = 0.0001). *p < 0.05; **p < 0.01; ***p < 0.001; ****p < 0.0001; *ns* not significant

To investigate the implication of endogenous SST in Hebbian LTP by a different approach, we used cysteamine, a compound that depletes SST levels in brain and other tissues [32, 33]. Mice received an intraperitoneal (IP) injection of cysteamine (150 mg/kg) and hippocampal slices were harvested 4 h later. The effect of cysteamine injection on SST levels in hippocampus was verified with immunofluorescence. SST immunofluorescence in CA1 SOM-INs expressing eYFP was decreased in cysteamine injected mice relative to vehicle injected mice (58.3 ± 9.7% of control; Fig. 3a), suggesting an effective lowering of SST levels in SOM-INs. Next, we examined Hebbian LTP in SOM-INs in slices of mice after vehicle or cysteamine IP injection. In vehicle injected mice, TBS elicited long-term potentiation of EPSP amplitude (145.8 ± 12.9% of control at 15–20 min, 147.5 ± 11.8% of control at 20–25 min and 160.7 ± 9.6% of control at 25–30 min) (Fig. 3b, c). In cysteamine injected mice, TBS failed to induce LTP of EPSP amplitude, but instead a slow onset depression of EPSPs was elicited (75.3 ± 7.7% of control at 15–20 min, 69.6 ± 8.7% at 20–25 min and 60.1 ± 8.5% of control at 25–30 min; Fig. 3b, c). Thus, lowering SST levels in SOM-INs by cysteamine IP injection, prevents Hebbian LTP induced by TBS.

To rule out extra-hippocampal effects of cysteamine IP injection, hippocampal slices from untreated mice were incubated in ACSF containing 200 µM cysteamine for 1 h. In cysteamine-treated slices, SST immunofluorescence was decreased in CA1 SOM-INs compared to vehicle-treated slices (76.9 ± 6.1% of control; Fig. 3d). Whole cell recordings showed that TBS failed to induce LTP of EPSP amplitude in SOM-INs of cysteamine-treated slices (89.2 ± 8.4% of control at 10–15 min, 89.4 ± 9.0% at 15–20 min and 83.3 ± 8.5% of control at 25–30 min). In contrast, TBS elicited LTP in SOM-INs of vehicle-treated slices (143.3 ± 14.8% of control at 10–15 min, 150.3 ± 14.0% of control at 15–20 min and 143.3 ± 9.1% of control at 25–30 min; Fig. 3e, f). Thus, lowering hippocampal SST levels interferes with Hebbian LTP induced by TBS in SOM-INs.

Taken together, the results of these experiments with cyclostomatostatine and cysteamine suggest that TBS may lead to the release of endogenous SST and the activation SSTRs in Hebbian LTP in SOM-INs.

### *SST*_*14*_* induced potentiation is independent of NMDAR and mGluR1a*

**Fig. 4:**
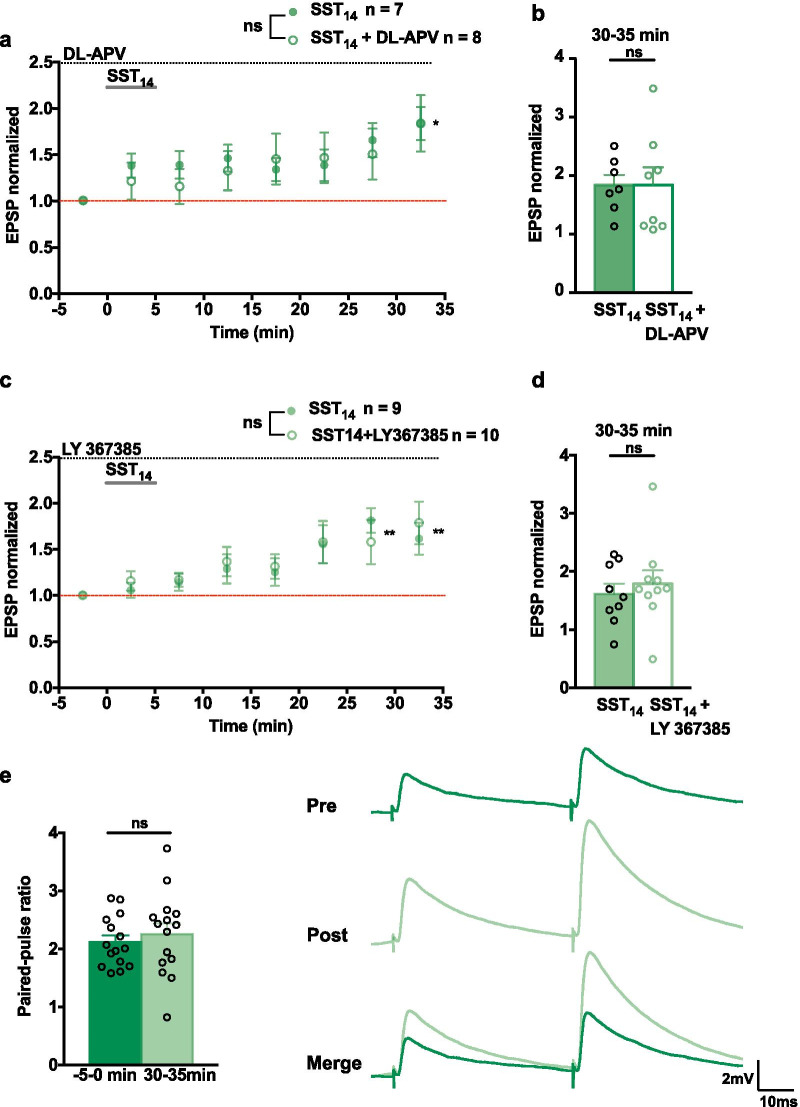
LTP induced by SST_14_ does not require NMDAR or mGluR1a. **a** Summary time plots of EPSP amplitude for experiments with application of SST_14_ in the presence (open green circle) and absence (filled green circle) of the NMDAR antagonist DL-APV (50 µM), indicating a similar slow onset LTP in both groups (SST_14_, n = 7 cells, 5 mice; SST_14_ + DL-APV, n = 8 cells, 6 mice; two-way mixed ANOVA *p* = 0.781, main effect of time *p* < 0.0001 with Bonferroni’s multiple comparisons at 30–35 min *p* = 0.016). **b** Summary bar graph of EPSP amplitude, showing similar LTP at 30–35 min post application. **c** Summary time plots of EPSP amplitude for experiments with application of SST_14_ in the presence (open green circle) and absence (filled green circle) of the mGluR1a antagonist LY367385 (40 µM), showing similar LTP in both groups (SST_14_, n = 9 cells, 7 mice; SST_14_ + LY367385, n = 10 cells, 7 mice; two-way mixed ANOVA *p* = 0.613, main effect of time *p* < 0.0001 with Bonferroni’s multiple comparisons at 25–30 min *p* = 0.004, 30–35 min *p* = 0.005). **d** Summary bar graph of EPSP amplitude indicating similar LTP at 30–35 min post application. **e** EPSPs from a representative cell receiving paired-pulse stimulation before and after SST_14_ application (right) and summary bar graph (left) showing similar paired-pulse ratio before (− 5 to 0 min) and after (30–35 min) SST_14_ application (n = 16 cells from SST_14_ groups in (**a**) and (**c**); paired *t*-test *p* = 0.531). *p < 0.05; **p < 0.01; *ns* not significant

NMDA receptors (NMDAR) and metabotropic glutamate 1a receptors (mGluR1a) are involved in synaptic plasticity in hippocampal interneurons [12, 34]. We examined a possible implication of these receptors in SST_14_-induced LTP in SOM-INs. First, we examined if NMDARs were implicated by including the antagonist DL-APV (50 µM) in the ACSF for the duration of the experiment. Bath application of SST_14_ in presence of DL-APV, induced a gradual LTP of EPSPs (183.9 ± 30.5% of control at 30–35 min) that was similar to the LTP elicited by SST_14_ without DL-APV (183.8 ± 17.8% of control at 30–35 min) (Fig. 4a, b). Thus, SST_14_-induced LTP does not require NMDARs.

Similarly, we examined the possible role of mGluR1a by including the antagonist LY367385 (40 µM) in the ACSF. Application of SST_14_ in the presence of LY367385 elicited LTP of EPSPs (178.8 ± 23.1% of control at 30–35 min) that was not different from the LTP induced by SST_14_ in the absence of LY367385 (161.5 ± 17.4% of control at 30-35 min) (Fig. 4c, d). In both groups, LTP developed gradually (EPSP amplitude 169.7 ± 3.3% of control at 25–30 min and 170.1 ± 3.4% of control at 30–35 min). Therefore, SST_14_-induced LTP does not involve mGluR1a.

In the above series of experiments with DL-APV and LY367385, we used paired stimulation to measure the paired-pulse ratio of EPSPs in cells that received SST_14_ in the absence of antagonists. Paired-pulse facilitation was similar before (-5 to 0 min; 2.116 ± 0.113) and after (30 to 35 min; 2.254 ± 0.184) SST_14_ application (Fig. 4e). These results suggest an absence of presynaptic changes during SST_14_-induced LTP.

**Fig. 5:**
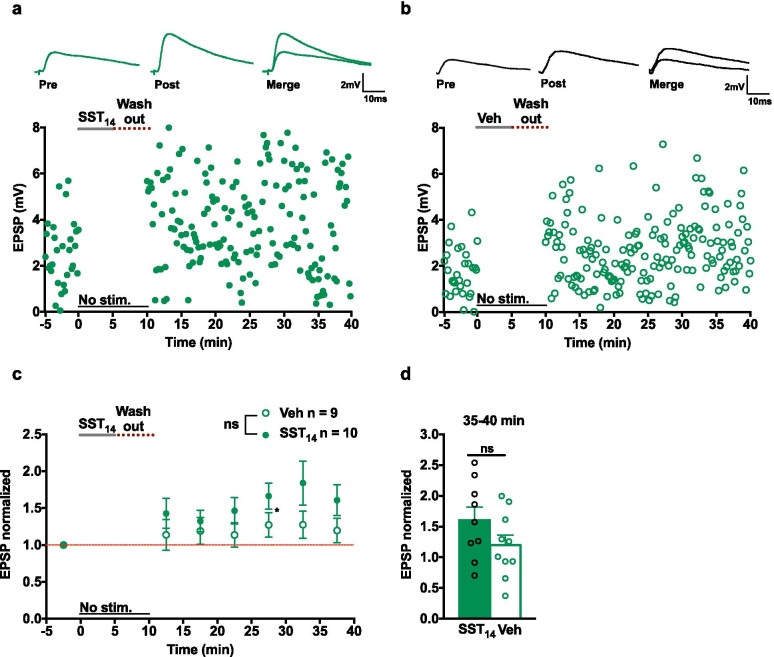
LTP of EPSPs is dependent on synaptic stimulation in the presence of SST_14_. **a**, **b** Examples of EPSPs (top) and summary time plots from representative cells receiving no synaptic stimulation during the 5 min SST_14_ (**a**) or vehicle (**b**) application and a 5 min wash-out. **c** Summary time plots of EPSPs for all cells showing a rebound potentiation of EPSP amplitude in both groups (Vehicle, n = 10 cells and 6 mice; SST_14_, n = 9 cells and 7 mice; two-way mixed ANOVA *p* = 0.258; main effect of time *p* = 0.004 with Bonferroni’s multiple comparisons 25–30 min *p* = 0.029). **d** Summary bar graph showing no difference in EPSP amplitude at 35–40 min after SST_14_ or vehicle application. *p < 0.05; ns: not significant

To shed further light on the mechanisms involved in the SST_14_-induced LTP, we examined if LTP is dependent on synaptic activity during SST_14_ application. EPSPs were recorded during a 5 min baseline period and stimulation was interrupted. SST_14_ was applied for 5 min and washed-out for another 5 min, without stimulation. Stimulation was resumed and EPSPs recorded for 30 min (Fig. 5a–c). Application of vehicle (127.2 ± 16.7% of control at 25–30 min) or SST_14_ (166.2 ± 17.8% of control at 25-30 min) produced similar effects with only a transient potentiation of EPSP amplitude (Fig. 5c). EPSP amplitude was not different at 35–40 min after application of vehicle (119.7 ± 16.6% of control) or SST_14_ (160.6 ± 21.1% of control) (Fig. 5c, d). These results suggest that, although interruption of stimulation induces a rebound potentiation in both groups, SST_14_-induced LTP of EPSPs may require synaptic stimulation in the presence of the peptide.

**Fig. 6:**
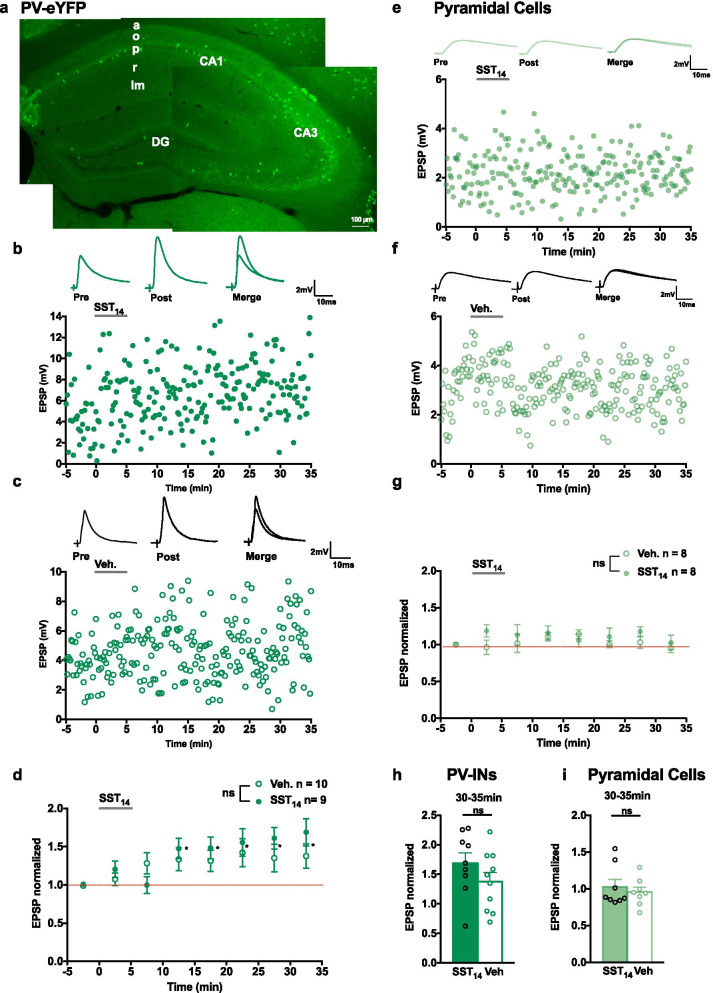
SST_14_ does not affect EPSPs in PV-INs or PCs. **a** Montage of fluorescence images showing eYFP expression in hippocampal PV-INs from PV-eYFP mouse. **b**, **c** Examples of EPSPs (top) and time plots of EPSP amplitude (bottom) from representative PV-INs receiving 5 µM SST_14_ (**b**) or vehicle (**c**). **d** Summary time plots of EPSPs (normalized to baseline) for all cells, showing that with application of either SST_14_ (filled green circle) or vehicle (open green circle), EPSPs showed a gradual run-up with no difference between vehicle and SST_14_ (Vehicle, n = 10 cells and 6 mice; SST_14_, n = 9 cells and 6 mice; two-way mixed ANOVA *p* = 0.141, main effect of time *p* = 0.0001 with Bonferroni’s multiple comparisons at 10–15 min *p* = 0.029, 15–20 min *p* = 0.027, 20–25 min *p* = 0.042, 25–30 min *p* = 0.021, 30–35 min *p* = 0.010). **e**–**g** Similar data presentation showing lack of effect of SST_14_ on EPSPs recorded in CA1 PCs. Examples of EPSPs (top) and time plots of EPSP amplitude (bottom) from representative cells receiving SST_14_ (**e**) or vehicle (**f**). Summary time plots of EPSPs for all cells (**g**), showing that SST_14_ (filled green square) or vehicle (open green circle) did not affect EPSPs in PCs. Two-way mixed ANOVA p = 0.506. **h**, **i** Bar graphs of EPSP amplitude in PV-INs (**h**) and PCs (**i**) at 30–35 min after SST_14_ or vehicle application showing no difference. *p < 0.05; *ns* not significant

The mGluR1a-dependent Hebbian LTP found at excitatory synapses onto SOM-INs is not observed at synapses onto PV-INs [8]. Therefore, we examined if application of SST_14_ affects EPSPs of PV-INs using a similar recording paradigm from PV-INs of PV-eYFP mice (Fig. 6a). After bath application of vehicle or SST_14_, EPSP amplitude increased similarly in both groups (140.4 ± 10.0% of control at 10–15 min; 139.9 ± 9.8% of control at 15–20 min; 148.8 ± 12.5% of control at 20–25 min; 148.1 ± 11.7% of control at 25–30 min; 153.2 ± 12.1% of control at 30-35 min; Fig. 6b–d). These results suggest that, under our recording conditions, EPSPs of PV-INs show spontaneous run-up over time. Moreover, application of SST_14_ produced similar results as vehicle (Fig. 6h), suggesting that SST_14_-induced potentiation may not occur at excitatory synapses onto PV-INs.

Excitatory synapses onto SOM-INs originate mostly from CA1 pyramidal cells (PCs). The actions of SST_14_ on CA1 PCs are complex and both inhibitory and excitatory effects of SST_14_ have been reported [22, 28, 35]. As SST_14_ effects on SOM-IN EPSPs could arise from indirect effects on PCs, we examined if SST_14_ affects EPSPs in PCs. EPSPs were recorded from CA1 PCs in slices from SOM-eYFP mice and SST_14_ was applied under similar conditions. Application of either SST_14_ or vehicle did not affect EPSP amplitude in PCs (SST_14_ group, 102.8 ± 10.0% of control at 30–35 min; vehicle group, 95.6 ± 6.6% of control at 30–35 min; Fig. 6e–g, i), indicating a lack of SST_14_ effect on PC EPSPs. These results suggest that SST_14_-induced LTP of EPSPs in SOM-INs may not arise from a di-synaptic effect via CA1 PC excitatory synapses.

### *SST actions on SOM-IN excitatory synapses mediated by GABA*_*A*_* inhibition*

**Fig. 7:**
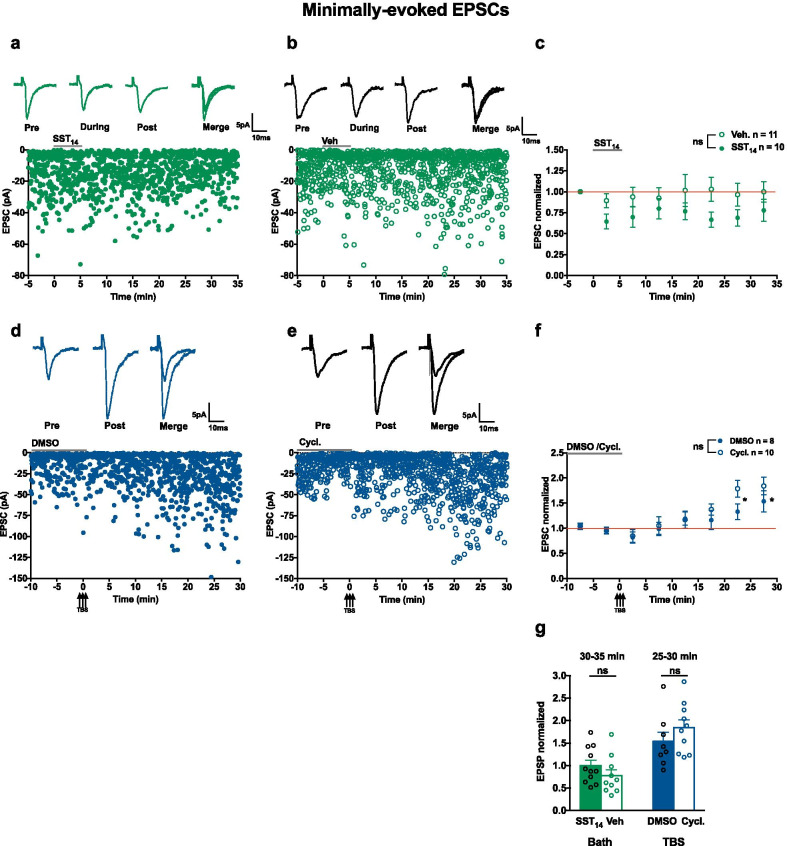
SST_14_ and cyclosomatostatin fail to affect pharmacologically isolated non-NMDAR-mediated EPSCs. **a**,** b** Voltage clamp recording of EPSCs (top) and time plots of EPSC amplitude from representative SOM-INs receiving 5 µM SST_14_ (**a**) or vehicle (**b**). EPSCs shown are average for -5 to 0 min baseline period (pre) and 30 to 35 min post-application period (post). **c** Summary time plots of EPSCs (normalized to baseline), showing lack of lasting effects on EPSC amplitude after SST_14_ application (filled green circle) or vehicle application (open green circle) (vehicle, n = 11 cells and 7 mice; SST_14_, n = 10 cells and 6 mice; two-way mixed ANOVA p = 0.485). **d**, **e** EPSCs (top) and time plots of EPSC amplitude from representative SOM-INs receiving TBS in the presence of DMSO (**d**) or 1 µM cyclosomatostatin (**e**). EPSCs shown are average for − 10 to 0 min baseline period (pre) and 25 to 30 min post-TBS period (post). **f** Summary time plots of EPSCs (normalized to baseline), showing similar LTP of EPSC amplitude induced by TBS in DMSO (filled blue circle) and cyclosomatostatin (open blue circle). For DMSO group, n = 9 cells and 7 mice, Two-way mixed ANOVA p = 0.402, main effect of time p < 0.0001 with Bonferroni’s multiple comparisons at 20–25 min *p* = 0.019, 25–30 min *p* = 0.017). **g** Summary bar graph showing no difference in EPSC amplitude at 30–35 min after SST_14_ or vehicle application (left), and increase in EPSC amplitude at 25–30 min after TBS showing similar LTP in DMSO and cyclosomatostatin (right). * p < 0.05; ns: no significant

To gain more insight on the mechanisms of SST_14_-induced LTP, we investigated the effects of SST_14_ on putative single fiber excitatory postsynaptic currents (EPSCs) evoked by minimal stimulation [12, 36]. Non-NMDAR-mediated EPSCs were recorded in whole cell voltage clamp mode from SOM-INs in the presence of NMDA and GABA_A_ receptor antagonists, DL-APV and gabazine respectively (Fig. 7a–c). EPSC amplitude was not affected by bath application of 5 µM SST_14_ (77.8 ± 13.3% of control at 30–35 min) or vehicle (99.9 ± 40.3% of control at 30-35 min) (Fig. 7c–g), suggesting a lack of lasting effect of SST_14_ on excitatory postsynaptic currents in these conditions.

Given this lack of effect of SST_14_ on EPSCs, we examined if the SST_1-5_R antagonist cyclosomatostatin affected TBS-induced LTP of EPSCs evoked by minimal stimulation. TBS given in the presence of DMSO elicited a slow onset LTP of EPSC amplitude (153.6 ± 20.9% of control at 30–35 min; Fig. 7d, f, g). Application of TBS in the presence of cyclosomatostatin produced LTP of EPSC amplitude that was not different from LTP elicited in DMSO (184.1 ± 17.6% of control at 30–35 min; Fig. 7e, f, g). These results indicate that blocking SST_1-5_Rs did not affect LTP of pharmacologically isolated non-NMDAR-mediated EPSCs.

**Fig. 8:**
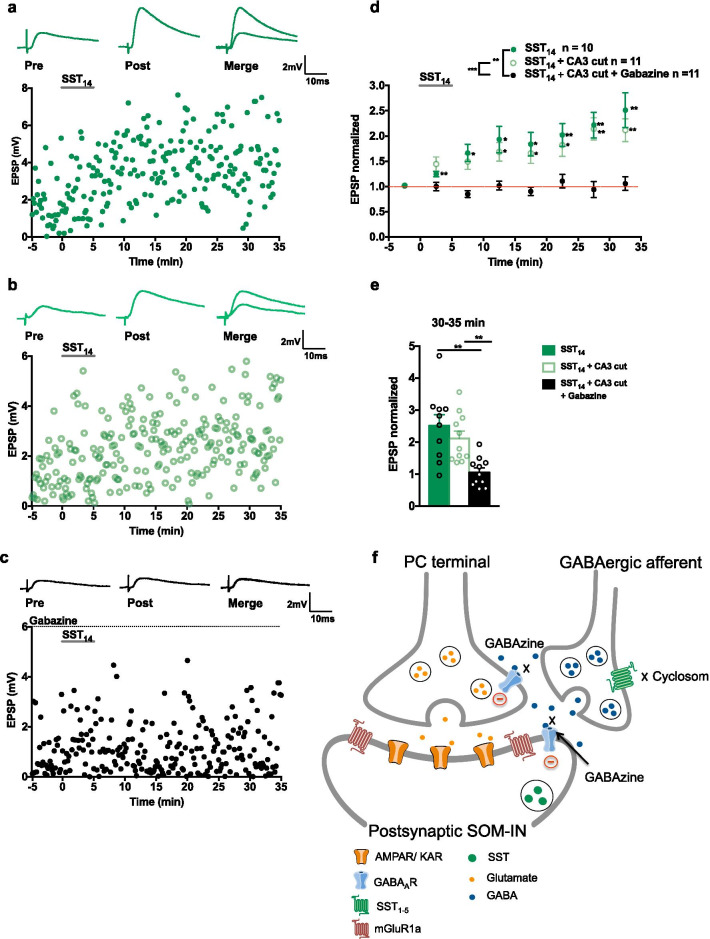
SST_14_-induced LTP of EPSPs is blocked by GABA_A_R antagonism. **a**–**c** EPSPs (top) and time plots of EPSP amplitude from representative SOM-INs receiving 5 µM SST_14_ in a normal slice (**a**), SST_14_ in a slice with a CA3-CA1 cut (**b**), or SST_14_ in a slice with a CA3-CA1 cut and the GABA_A_ receptor antagonist gabazine (5 µM) (**c**). **d** Summary time plots of EPSPs (normalized to baseline) for all cells, showing LTP of EPSPs after SST_14_ application in normal slices (filled green circle) and in slices with a CA3-CA1 cut (open green circle), but not after SST_14_ application in the presence of gabazine in slices with a CA3-CA1 cut (black). For SST_14_ in normal slices; n = 10 cells and 7 mice; rmANOVA with Dunnett’s multiple comparisons (0–5 min *p* = 0.008, 5–10 min *p* = 0.023, 10–15 min *p* = 0.033, 15–20 min *p* = 0.033, 20–25 min *p* = 0.009, 25–30 min *p* = 0.007, 30–35 min *p* = 0.009). For SST_14_ in slices with CA3 cut; n = 11 cells and 6 mice; rmANOVA with Dunnett’s multiples comparisons (10–15 min *p* = 0.023, 15–20 min *p* = 0.046, 20–25 min *p* = 0.027, 25–35 min *p* = 0.003, 30–35 min *p* = 0.004). For SST_14_ in gabazine; n = 11 cells and 6 mice; rmANOVA *p* = 0.974. **e** Summary bar graph of EPSP amplitude at 30–35 min, showing absence of long-term changes in EPSP amplitude induced by SST_14_ in the presence of gabazine. SST_14_ *vs* SST_14_ in gabazine comparison, two-way mixed ANOVA, univariate analysis at 30–35 min *p* = 0.001. SST_14_ with CA3 cut *vs* SST_14_ with CA3 cut and gabazine comparison, two-way mixed ANOVA, univariate analysis at 30–35 min *p* = 0.001. **f** Model of the mechanism of SST actions in LTP of excitatory synapses onto SOM-INs (details in text). *p < 0.05; **p < 0.01

As the non-NMDAR-mediated EPSCs in the previous experiments were pharmacologically isolated in the presence of the GABA_A_ receptor antagonist gabazine and in slices with CA3-CA1 surgical cuts, we examined if the long-lasting effects of SST_14_ on EPSPs was due to an indirect action via GABA_A_ receptors. We tested the effects of SST_14_ on EPSPs of SOM-INs in normal slices, slices with a CA3-CA1 cut, or slices with a CA3-CA1 cut and gabazine (Fig. 8). Application of SST_14_ in normal slices evoked a gradual long-term increase in EPSP amplitude (124.4 ± 5.1% of control at 0–5 min, 166.3 ± 17.0% of control at 5–10 min, 193.3 ± 26.2% of control at 10–15 min, 187.3 ± 23.2% of control at 15–20 min, 202.0 ± 22.7 of control at 20–25 min, 221.8 ± 25.4% of control at 25–30 min, 251.2 ± 34.2% of control at 30–35 min; Fig. 8a, d, e). SST_14_ application in slices with a CA3-CA1 cut produced a similar long-lasting potentiation of EPSP amplitude (168.5 ± 18.4% of control at 10–15 min, 165.8 ± 19.8% of control at 15–20 min, 182.1 ± 22.5% of control at 20–25 min, 214.1 ± 22.1% of control at 25–30 min, 211.7 ± 22.8% of control at 30–35 min; Fig. 8b, d, e). In contrast, SST_14_ application in slices with a CA3-CA1 surgical cut and in the presence of gabazine did not affect EPSP amplitude (94.0 ± 15.6% of control at 25–30 min, 105.5 ± 13.4% of control at 30–35 min; Fig. 8c, d, e), indicating that antagonism of GABA_A_ receptors blocked the SST_14_-induced potentiation of EPSPs. Thus, SST actions in LTP of excitatory synapses of SOM-INs may be indirectly mediated via GABA_A_ inhibition (Fig. 8 f).

## Discussion

In the present work, we uncover a critical involvement of the peptide SST in long-term potentiation at excitatory synapses of hippocampal SOM-INs. We found that application of exogenous SST_14_ induces long-term potentiation of EPSPs in SOM-INs via somatostatin type 1–5 receptors (SST_1-5_Rs) (Fig. 1). Application of SST_14_ did not affect EPSPs in PCs or parvalbumin-expressing interneurons (Fig. 6). TBS-induced Hebbian LTP of EPSPs in SOM-INs was prevented by inhibition of SST_1-5_Rs (Fig. 2) and by depletion of hippocampal SST by cysteamine treatment (Fig. 3), suggesting a significant role of endogenous SST in LTP. LTP of SOM-IN EPSPs induced by SST_14_ did not involve changes in paired-pulse ratio of synaptic responses (Fig. 4), was independent of NMDAR and mGluR1a (Fig. 4) and was dependent on concomitant synaptic activity (Fig. 5). Importantly, we observed that SST_14_ did not affect non-NMDAR-mediated EPSCs recorded during GABA_A_ receptor blockade, and that the SST_1-5_R antagonist cyclosomatostatin did not affect TBS-induced LTP of these EPSCs (Fig. 7). Finally, pharmacological block GABA_A_ receptor function prevented SST_14_-induced potentiation of EPSPs (Fig. 8), indicating that SST_14_ long-term potentiation of excitatory synaptic responses is an indirect effect via GABA_A_ inhibition.

Our results suggest the following model of the contribution of endogenous SST in Hebbian LTP at excitatory synapses of SOM-INs (Fig. 8f): theta burst stimulation of PC axons (i) releases glutamate at PC synapse onto SOM-IN, (ii) eliciting EPSP and action potential firing in SOM-IN, (iii) leading to SST release from SOM-IN, (iv) activation of SSTRs on GABAergic afferent, (v) inhibition of GABA release, (vi) disinhibition of pre- or postsynaptic compartment of the glutamatergic synapse, and (vii) potentiation of EPSP in SOM-IN. Our findings uncover a novel role for SST in long-term plasticity of excitatory synapses onto somatostatinergic cells by indirectly regulating GABA_A_ inhibition.

### *SST*_*14*_*-induced LTP of SOM-INs excitatory synapses*

The observed long-lasting potentiation of EPSPs in SOM-INs by SST_14_ contrasts with the previously described SST actions in hippocampal PCs. Bath-applied SST_14_ was reported to decrease evoked and spontaneous EPSCs in pyramidal cells [25]. These effects were acute, occurring within 2–4 min of application onset, and rapidly (2–4 min) reversible. Consistent with these previous observations, we did not find long-lasting effects of SST_14_ on EPSPs in PCs, suggesting that SST_14_ long-term effects on SOM-IN excitatory synapses do not arise from di-synaptic actions at PC afferent synapses, but are specific to synapses onto interneurons. Moreover, SST_14_ did not affect excitatory synaptic responses of PV-INs, indicating that SST_14_ long-term effects may be specific to excitatory synapses onto somatostatinergic cells. However, in experiments with recordings from PV-INs, the same basal recording and stimulating conditions that elicited stable synaptic responses in SOM-INs and pyramidal cells, resulted in a spontaneous run-up over time. Previous work has shown that excitatory synapses of PV-INs display an anti-Hebbian form of LTP mediated by Ca^2+^-permeable AMPARs [34]. Since in our recording conditions we maintained the postsynaptic PV-IN at hyperpolarized level during stimulation, the run-up over time may have been caused by anti-Hebbian plasticity. But anti-Hebbian plasticity is independent of GABA_A_ inhibition [34], and, thus, unlikely to occlude SST_14_ actions via GABA_A_ inhibition. Thus, it would be important to re-examine the SST_14_ effects on PV-INs excitatory synapses using different recording/stimulation conditions that elicit stable basal responses, to rule out a possible occlusion of SST_14_ effects by the response run-up over time. Hippocampal PCs are also hyperpolarized by bath application of SST_14_ via activation of postsynaptic K^+^ conductances [22–24]. These are also acute effects, reversible in minutes, that are unlikely to contribute to the slow onset and long-lasting potentiation of EPSPs in SOM-INs. Furthermore, SST_14_-induced hyperpolarization of PCs reduces action potential firing [22], and thus would be expected to reduce presynaptic activation of PCs and decrease EPSPs in SOM-INs. Although SST_14_ has been reported to depolarize and excite PCs [35], these effects are produced by local application of SST_14_ and are not observed with bath application [24]. Thus, these direct membrane effects of SST_14_ on PCs are distinct from the long-lasting actions of SST_14_ on SOM-IN excitatory synapses observed here.

### *SST*_*14*_*-induced potentiation is mediated by GABA*_*A*_* inhibition*

SST_14_-induced potentiation of EPSPs was prevented by the GABA_A_ antagonist gabazine, indicating that SST_14_ actions are mediated indirectly via GABA_A_ inhibition. The mechanism by which SSTR activation acts via GABA_A_ inhibition to increase synaptic excitation remains to be clarified. However, SSTRs are coupled to presynaptic inhibition via inhibition of voltage-gated Ca^2+^ channels and activation of K^+^ currents [17, 18]. Thus, via such mechanisms, activation of SSTRs could inhibit release from GABAergic terminals, blocking GABA_A_ inhibition at the PC synapse onto SOM-IN, resulting in potentiation at this synapse via disinhibition. Inhibition of GABAergic synaptic transmission in PC was reported by local application of SST_14_ [28]. However, bath application of SST_14_ was reported to inhibit selectively synaptic excitation without affecting inhibition in PCs [25]. SST_14_ actions in hippocampal interneurons also appear complex with report of depolarization and hyperpolarization [35]. In other brain regions, SST was found to decrease GABA release [17].

Whether SST actions are mediated via GABA_A_ inhibition acting pre- or post-synaptically at the PC to SOM-IN synapse remains unclear. Paired-pulse ratio was unchanged during and after SST_14_ application (Fig. 4), suggesting no presynaptic changes in transmitter release. However, the excitatory synapses from PC to SOM-INs are composed of calcium-permeable AMPA receptors, and paired stimulation of synaptic responses can be affected also by postsynaptic AMPAR mechanisms [36]. Thus, a lack of change in paired-pulse ratio may not be a reliable indication of a lack of pre-synaptic GABA_A_ inhibition at these synapses. Further experimentation assessing additional parameters, such as coefficient of variation of synaptic currents [37], may be useful to help resolve this issue. Another possibility to assess if the SST-mediated indirect GABA_A_ receptor inhibition occurs only at the presynaptic PC terminal would be to observe if the NMDA-component of synaptic responses recorded from SOM-INs is also potentiated by SST_14_.

SOM-INs receive postsynaptic GABA_A_ mediated inhibition [13], notably from interneuron-selective interneurons expressing vasoactive intestinal peptide [38, 39], and these could be targeted by SST. However, we did not observe changes in cell input resistance during and after SST_14_ application (Fig. 1), suggesting no postsynaptic change. However, cell input resistance was measured at the soma and synaptic inhibition may occur at more remote dendritic sites [39]. Interestingly, local application of somatostatin depresses inhibitory postsynaptic potentials recorded in CA1 pyramidal cells, without affecting postsynaptic responses to exogenously applied GABA, indicating somatostatin-induced presynaptic inhibition of GABA synaptic responses in pyramidal cells [28]. Likewise, bath application of somatostatin presynaptically inhibits GABA synaptic transmission onto basal forebrain cholinergic neurons [29]. Similar experiments on inhibitory synaptic transmission onto SOM-INs would be important to clarify the mechanisms of SST_14_ actions via GABA_A_ inhibition. Our results also raise the question of which type of GABAergic interneuron expresses SSTRs pre-synaptically? SST_1-4_R, and to a lesser extent SST_5_R, are present in CA1 hippocampus and in pyramidal cells [40–42]. Generally, SSTR subtypes preferentially occupy specific cell compartments. SST_1_R is mainly pre-synaptic, SST_2,4,5_R post-synaptic, and SST_3_R extra-synaptic (neuronal cilia) [43]. However, which inhibitory cell type in the hippocampus expresses the receptors and whether they are pre- or post-synaptic remains largely to be determined [42, 43]. Interestingly, SST_5_R and CB1 receptors co-localize in some CA1 interneurons [21]. CB1 receptors are highly expressed mostly in inhibitory interneurons that co-express the neuropeptide cholecystokinin (CCK) [44], suggesting that these interneuron subtypes may mediate SST actions on presynaptic GABA_A_ inhibition. Further experiments will be required to elucidate the pre- and/or post-synaptic GABA_A_ mechanisms involved in the disinhibitory actions of SST at SOM-IN excitatory synapses.

Our results with the antagonist cyclosomatostatin are also consistent with an effect of endogenous SST released after theta burst stimulation contributing to long-term potentiation at the PC to SOM-IN synapse indirectly via GABA_A_ inhibition. Application of SST_14_ failed to modify non-NMDAR-mediated EPSCs recorded in the presence of gabazine (Fig. 7). In addition, the SSTR antagonist cyclosomatostatin did not affect TBS-induced LTP of non-NMDAR-mediated EPSCs recorded in the presence of gabazine (Fig. 7). However, SST_14_ and cyclosomatostatin showed effects on EPSPs recorded with GABA_A_ inhibition intact (Figs. 1 and 2). These results suggest that, under physiologically relevant conditions, release of endogenous SST by theta burst stimulation contributes to long-term potentiation at PC to SOM-IN synapses indirectly via GABA_A_ inhibition.

### Mechanisms of SST-induced LTP

Although synaptic plasticity in some hippocampal interneurons involves NMDARs [34], mGluR1a-mediated Hebbian LTP in SOM-INs does not [12]. Consistent with this notion, our results indicate that SST_14_-induced LTP in SOM-INs is unaffected by the NMDAR antagonist DL-APV, and thus does not involve NMDARs.

Hebbian LTP requires mGluR1a activation [12] and our results indicate that SST_14_ actions that lead to LTP of EPSPs occur downstream of mGluR1a activation since the antagonist LY367385 does not prevent SST_14_-induced potentiation. Moreover, the long-lasting actions of SST_14_ are activity-dependent and require concomitant synaptic activity during application of SST_14_. In these experiments we observed that when synaptic stimulation was re-initiated after a period of interruption a rebound potentiation of synaptic responses was observed. Previous work in oriens-alveus interneurons has shown that, during recordings with intracellular BAPTA to buffer postsynaptic Ca^2+^ levels, LTP is blocked [37]. However, in addition, the injection of BAPTA induces a long-lasting depression of synaptic responses [37]. Thus, postsynaptic Ca^2+^ mechanisms are necessary for LTP induction and for maintenance of intact transmission at these synapses. Moreover, activation of excitatory synapses of SOM-INs involve calcium-permeable AMPARs [36] and Ca^2+^ influx [45]. These results suggest that inactivation and subsequent reactivation of synapses may influence postsynaptic Ca^2+^ homeostasis, resulting in rebound potentiation. Importantly, in comparison to SST-induced LTP, the magnitude of rebound potentiation was variable and transient, only reaching significance at 15–20 min after resuming stimulation, suggesting different mechanisms at play. Since SST-induced potentiation via GABA_A_ inhibition occurs downstream of AMPAR- and mGluR1a-mediated Ca^2+^ signals, the mechanisms of rebound potentiation are unlikely to have interfered with, or occluded, the SST effects. Thus, SST_14_ actions on GABA_A_ inhibition may require synaptic activity during SSTR activation to lead to long-lasting changes. Intriguingly, long-lasting reduction of synaptic inhibition by local application of SST was previously reported in PCs [28] but not with bath application [25]. Further experiments focusing on GABA_A_ inhibition of SOM-INs will be necessary to explain the activity-dependent disinhibitory actions of SST_14_ in SOM-INs.

### Endogenous SST contributes to mGluR1a-mediated Hebbian LTP

Our results with the SST_1-5_R antagonist cyclosomatostatin suggest that, under physiologically relevant conditions, theta burst stimulation causes release of endogenous SST which contributes to LTP at SOM-IN excitatory synapses via GABA_A_ disinhibition. The release of endogenous SST is frequency-dependent, as EPSPs elicited at 0.1 Hz are unaffected by the antagonist (Fig. 1). During the LTP induction protocol, theta burst stimulation elicits EPSPs that cause action potential burst firing in SOM-INs [8], conditions that are sufficient to cause release of endogenous SST. Such an activity-dependent release of SST is consistent with recent evidence that release of endogenous SST in acute prefrontal cortex slices is induced by frequency-dependent (> 10 Hz) optogenetic stimulation of SOM-INs [46] and that release of endogenous somatostatin in cultured hippocampal neurons is stimulated by AMPA receptor activation [47].

Our results with SST depletion by cysteamine also support a role of endogenous SST in Hebbian LTP. We found that systemic injection of cysteamine or in vitro treatment of slices with cysteamine prevented TBS-induced Hebbian LTP, providing further support for a role of release of endogenous SST in LTP at SOM-IN excitatory synapses. We observed that TBS resulted in long-lasting depression of EPSPs after systemic cysteamine treatment. Since no lasting depression was observed after in vitro treatment of slices with cysteamine, the depression may be the result of extra-hippocampal effects of cysteamine treatment. In previous work during recordings with intracellular BAPTA to prevent postsynaptic Ca^2+^ rise, LTP was blocked in oriens-alveus interneurons and replaced by LTD [37]. Moreover, in this previous work, injection of BAPTA alone induced a long-lasting decrease in EPSC amplitude, indicating that postsynaptic Ca^2+^ mechanisms are necessary for LTP induction and for maintenance of intact transmission at these synapses [37]. Thus, extra-hippocampal effects of systemic cysteamine treatment may have interfered with Ca^2+^ homeostasis in SOM-INs and resulted in LTD.

SST was previously shown to be critical for hippocampal long-term synaptic plasticity, as well as learning and memory. Depletion of SST by cysteamine treatment, or knock-out of the SST gene in transgenic mice, impairs hippocampus-dependent contextual fear memory but not hippocampus-independent auditory fear learning [30]. The memory impairment is associated with a decrease in LTP in CA1 PCs [30], as well as at mossy-fiber CA3 PC synapses [48]. SST-induced LTP in SOM-INs may be the link between the role of SST in regulation of hippocampal network plasticity and hippocampal memory. Firstly, contextual fear learning was shown to induce a persistent LTP at excitatory synapses of SOM interneurons mediated by mGluR1 and mTORC1 [9]. Our finding that SST contributes to mGluR1a-mediated Hebbian LTP in SOM-INs, suggests that SST-induced LTP may be induced by contextual learning. Secondly, SOM cell-specific transgenic mouse approaches have shown a functional role of LTP at SOM interneuron excitatory synapses in hippocampal learning and memory [9]. Genetic down-regulation of mTORC1 activity impaired, whereas up-regulation facilitated, mGluR1a-mediated LTP at SOM interneurons excitatory synapses [9]. At the network level, SOM interneurons, and most notably OLM cells, are dendrite projecting inhibitory interneurons that differentially regulate Schaffer collateral (SC) and temporo-ammonic (TA) pathways onto CA1 pyramidal cells [7]: they suppress the distal TA pathway and facilitate the more proximal SC pathway [7]. Thus, LTP at excitatory synapses onto SOM interneurons causes long-term changes in their output firing [36] and inhibition of pyramidal cells [37], resulting in differential long-term regulation of plasticity at SC and TA synapses onto pyramidal cells: up-regulation of plasticity of the SC pathway [8, 9] and down-regulation of plasticity of the TA pathway [11]. At the behavioral level, genetic loss of mTORC1 function specifically in SOM interneurons impaired contextual fear and spatial long-term memories, whereas genetic upregulation of mTORC1 augmented spatial and contextual fear memories [9]. Thus, learning-induced LTP at SOM-IN excitatory synapses is linked to regulation of CA1 network metaplasticity and hippocampal long-term memory consolidation [9]. Our findings that endogenous SST plays a critical role in LTP at SOM-IN excitatory synapses, suggest that impairments in LTP in CA1 pyramidal cells and deficits in contextual fear memory caused by SST depletion/knockout [30] may be due to loss of SST-mediated LTP at SOM-IN synapses [9]. Thus, the role of SST in long-term synaptic plasticity of SOM-INs uncovered here may be crucially implicated in SST regulation of hippocampal learning and memory.

## Methods

### Animals

All animal procedures and experiments were performed in accordance with Université de Montréal Animal Care Committee (Comité de déontologie de l'expérimentation sur les animaux, CDEA) and followed the guidelines of the Canadian Council on Animal Care. Experiments were carried out on mice (5–8 week-old males for electrophysiology, and from both sexes for immunofluorescence). Mice were housed 2–5 per cage and given ad libitum access to food and water, in temperature (~ 22 °C) and humidity (~ 55%) controlled rooms with a normal 12 h light/dark cycle.

### Transgenic mice lines

Transgenic mice expressing Cre-dependent enhanced yellow fluorescent protein (eYFP) in SOM-INs (SOM-eYFP mice) were generated by crossing a knock-in mouse with an internal ribosome entry site (IRES)-linked Cre recombinase gene downstream of the *Sst* locus (*Sst*^ires−Cre^; The Jackson laboratory, Bar Harbour, ME JAX #013044) with *Rosa26*^lsl−EYFP^ reporter mice (Ai3; JAX #007903). Mice expressing eYFP in parvalbumin interneurons (PV-eYFP mice) were generated by crossing *Pvalb*^ires−Cre^ mice (JAX #008069) with *Rosa26*^lsl−EYFP^ reporter mice (Ai3; JAX #007903).

### Cysteamine injection

To study somatostatin depletion, SOM-eYFP mice were injected intraperitoneally (IP) with 150 mg/kg cysteamine (Sigma-Aldrich; M6500) diluted in bacteriostatic NaCl 0.9% (Hospira) or vehicle [49]. After 4 h, mice were anaesthetized with isoflurane inhalation and then decapitated to obtain acute hippocampal slices, as described below. Some mice were deeply anesthetized with sodium pentobarbital (MTC Pharmaceuticals, Cambridge, Ontario, Canada) and were perfused transcardially, first with ice-cold 0.1 M phosphate buffer (PB), then with 4% para-formaldehyde in 0.1 M PB (PFA) and the brain isolated. Post-fixed brains were cryoprotected in 30% sucrose and coronal brain sections (50 µm thick) were obtained for immunofluorescence.

### Acute hippocampal slice preparation

SOM-eYFP or PV-eYFP mice were anaesthetized with isoflurane inhalation and then decapitated. The brain was rapidly removed and placed in ice-cold sucrose-based solution containing (in mM): 75 sucrose, 87 NaCl, 2.5 KCl, 1.25 NaH_2_PO_4_, 7 MgSO_4_, 0.5 CaCl_2_, 11.6 ascorbic acid, 3.1 pyruvic acid, 25 D-glucose et 25 NaHCO_3_ (pH 7.3 ± 0.05; 300 ± 5 mOsmol/L). A block of tissue containing the hippocampus was obtained from each hemisphere and 300 µm thick transverse hippocampal slices were prepared with a Leica VT1000S vibratome. Slices were transferred for a 30 min recovery period in artificial cerebrospinal fluid (ACSF) containing the following (in mM) 124 NaCl, 2.5 KCl, 1.25 NaH_2_PO_4_, 26 NaHCO_3_, 1.3 MgSO_4_,10 D-glucose, 2.5 CaCl_2_ (pH 7.3–7.4, 295–305 mOsmol/L) at 30 °C and subsequently maintained at room temperature (20 –22 °C) for at least 60 min, until use. Both cutting solution and ACSF were saturated with 95% O_2_/5% CO_2_.

### Cysteamine treated acute hippocampal slices

Hippocampal slices were obtained as above and transferred in oxygenated ACSF containing cysteamine (200 µM) or ACSF alone, for 1 h at room temperature. Slices were then used for electrophysiological whole cell recording (as described below) or fixed overnight at 4 °C with 4% PFA, rinsed with PB 0.1 M, cryoprotected in 30% sucrose/PB 0.1 M and re-sectioned (50 µm) using a freezing microtome (Leica SM200R, Germany) for immunofluorescence.

### Whole-cell current clamp recordings

Slices were transferred to a submersion chamber perfused with ACSF (3–4 ml/min) at 31 ± 0.5˚C. CA1 interneurons expressing eYFP, or pyramidal cells not expressing eYFP, were identified using an upright microscope (Zeiss Axioskop, Toronto, Canada) with a water-immersion long-working distance objective (40X N-Achroplan, Zeiss, Toronto, Canada), epifluorescence lamp (FluoArc N HBO 103, Zeiss, Toronto, Canada) and an infrared digital video camera (Infinity 3, Lumenera, Ottawa, Canada). Whole-cell current clamp recordings were obtained using borosilicate glass pipettes (2–5MΩ; WPI) filled with intracellular solution containing (in mM): 120 KMeSO_4_, 10 KCl, 10 HEPES, 0.5 EGTA, 10 Na_2_-phosphocreatine, 2.5 MgATP, 0.3 NaGTP (pH 7.4, 300 mOsmol/L). Data were acquired using a Multiclamp 700B amplifier (Molecular Devices), digitized at 20 kHz using Digidata 1440A and pClamp 10 (Molecular Devices). Recordings were low-pass filtered at 2 kHz. Access resistance was regularly monitored during experiments and data was included only if the holding current was stable and access resistance varied less than 20% of initial value. Excitatory postsynaptic potentials (EPSPs) were evoked using constant current pulses (50 µs duration) via a concentric bipolar Pt/Ir electrode (FHC) placed in stratum oriens near the alveus, 100 µm lateral from the recorded cell soma. Membrane potential was held at -60 mV by constant current injection. EPSPs were evoked during a hyperpolarizing current step (5–10 mV, 0.5–1 s duration) to avoid action potential generation. Paired stimulations (50 ms inter-event interval) were given at 0.1 Hz. LTP was induced by three episodes (at 30 s intervals) of theta-burst stimulation (TBS) of afferents (five bursts, each consisting of four pulses at 100 Hz, with a 250 ms interburst interval).

### Whole-cell voltage clamp recordings

The protocol for slice preparation was as described above, except that CA1-CA3 regions were disconnected by a surgical cut. Glass pipettes were filled with intracellular solution containing (in mM): 120 CsMeSO_3_, 5 CsCl, 2 MgCl_2_, 10 HEPES, 0.5 EGTA, 10 Na_2_-phosphocreatine, 2 ATP-Tris, 0.4 GTP-Tris, 0.1 spermine, 2 QX314 (pH 7.2–7.3; 280 ± 5 mOsmol). Excitatory postsynaptic currents (EPSCs) were evoked using constant current pulses (50 µs duration) via an ACSF-filled bipolar theta-glass electrode (Harvard Apparatus) positioned 100 µm lateral to the recorded cell soma at the border between CA1 stratum oriens and the alveus. EPSCs were evoked at 0.5 Hz using minimal stimulation adjusted to obtain approximately 50% success events and 50% failures (paired stimulation with 50 ms interval). EPSCs were recorded in the presence of DL-amino-5-phosphonovaleric acid (APV; 50 µM, abcam #120004) and SR-95531 (gabazine, 5 µM; abcam #120042) to block NMDA and GABA_A_ receptors, respectively. For experiments with TBS-induced LTP of EPSCs, the ACSF contained 4 mM Mg^2+^ and Ca^2+^ to reduce spontaneous EPSC activity [12]. EPSCs and EPSPs were usually characterized in one cell per slice, and the different experimental conditions were interleaved. Responses were analyzed off-line using Clampfit (pClamp 10; Molecular Devices), GraphPad (Prism 7.2), SPSS 26 (IBM). Amplitude of EPSP and EPSC (average peak response; including failures for EPSCs) were averaged in 5 min bins over the total 35–40 min period of recordings.

### Somatostatin immunofluorescence

Sections were permeabilized with 0.3% Triton X-100 in 0.1 M PB (15 min) and unspecific binding was blocked with 10% normal goat serum in 0.1% Triton X-100 and 0.1 M PB (1 h). Sections were incubated 24–48 h at 4ºC with mouse monoclonal somatostatin antibody (1/500; Santa Cruz Biotechnology; Dallas, TX), and subsequently at room temperature with Rhodamine-Red™ X-conjugated goat anti-mouse IgG2b (1/200; 90 min; Jackson Immunoresearch Labs; West Grove, PA). Sections were mounted in ProLong™ Diamond (Life technologies; Carlsbad, CA) and images were acquired using a confocal microscope (LSM880; Carl Zeiss, Oberkochen, Germany) at excitation 488 and 543 nm. Images were acquired using the exact same parameters fixed on control slices (ACSF) or mice (saline). The intensity of the somatostatin immunofluorescence, in *oriens-alveus* region of the CA1 hippocampus was quantified using ImageJ software (National Institute of Health; https://github.com/imagej/imagej1) by comparing integrated density in cells corrected for background fluorescence. For experiments with cysteamine IP injection, cell fluorescence was measured typically in 44–63 fields of view per animal coming from 3–4 sections and averaged per animal. A total of 3 animals per group coming from 3 independent experiments were analyzed (total of 397 cells for saline; 441 cells for cysteamine). For acute slices, cell fluorescence was measured typically in 3–49 fields of view per slice coming from 2–4 sections and averaged per slice. A total of 3 animals coming from 3 independent experiments were analyzed (total of 714 cells for ACSF; 713 cells for cysteamine).

### Pharmacology

The neuropeptide somatostatin (SST_14_; Abcam #141206) was diluted daily in ACSF at 5 µM and perfused for 5 min and then washed-out during whole cell recordings of EPSPs/EPSCs. In some experiments, cyclosomatostatin (Abcam #141211), a non-selective SST_1-5_R antagonist was dissolved in DMSO and applied at a final concentration of 1 µM in ACSF. It was perfused for 5 min before and during somatostatin application, and then washed out. In TBS LTP experiments, cyclostomatostatin was perfused for 10 min before and during TBS, and then washed out. In some experiments, 40 µM LY367385 (Tocris #1237), a mGluR1a selective antagonist, or 50 µM DL-APV (Abcam #120004), a NMDAR selective antagonist, were diluted in ACSF and perfused throughout the recording period (40 min).

### Statistical analysis

No statistical methods were used to predetermine sample size but our sample sizes are comparable to those used generally in the field. Statistical analysis was performed using SPSS statistics 26 (IBM). For experiments with two groups with repeated measures in each group, a two-way mixed ANOVA was performed. Outliers were removed if values of studentized residuals were greater than ± 3. Normality of data distribution was validated by Skewness and Kurtosis values. The assumption of homogeneity of variance was assessed by Levene’s test of equality of error variances. The assumption of sphericity was assessed by Mauchly’s test of sphericity. If the assumption of sphericity was violated, a Greenhouse–Geisser correction was applied. If two-way ANOVA showed a significant interaction between group and time, the interaction was decomposed with a univariate analysis of variance and one-way repeated measure ANOVA (rmANOVA). Univariate analysis of variance was used to compare between groups at each time point. rmANOVA with Dunnett’s multiple comparisons was used to compare each time point inside the same group to baseline. If no statistical interaction was found, only the main effect of group and time was reported with Bonferroni adjustment for multiple comparisons. In the figures, data are expressed as arithmetic mean ± SEM. Asterisks denote statistical significance as calculated by the specified statistical tests (*p < 0.05; **p < 0.01; ***p < 0.001; ****p < 0.0001; ns, not significant). Detailed results of all statistical tests referenced per figure are included in a supplemental table (Additional file 1).

## Supplementary Information

**Additional file 1.** Statistical table**.** Description of data: results of all statistical tests referenced per figure.

## Data Availability

The datasets used and/or analysed during the current study are available from the corresponding author on reasonable request.

## Abbreviations

ACSF: Artificial cerebrospinal fluid
AMPAR: α-Amino-3-hydroxy-5-methyl-4-isoxazolepropionic acid receptor
APV: DL-amino-5-phosphonovaleric acid
eIF2α: Eukaryotic initiation factor 2α
EPSC: Excitatory postsynaptic current
EPSP: Excitatory postsynaptic potential
eYFP: Enhanced yellow fluorescent protein
GABA_A_: γ-Aminobutyric acid type A
IP: Intraperitoneal injection
LTP: Long-term potentiation
mGluR1a: Metabotropic glutamate receptors type 1a
mTORC1: Mammalian/mechanistic target of rapamycin complex 1
NMDAR: N-methyl-D-aspartate receptor
O-LM: Oriens-lacunosum/moleculare
PB: Phosphate buffer
PC: Pyramidal cell
PV-IN: Parvalbumin-expressing interneuron
SOM-IN: Somatostatin-expressing interneuron
SRIF: Somatotropin-release inhibitory factor
SST: Somatostatin
SST_14_: Somatostatin-14
SST_1-5_R: Somatostatin type 1–5 receptor
TBS: Theta-burst stimulation
