# Supplementary material for: Somatostatin contributes to long-term potentiation at excitatory synapses onto hippocampal somatostatinergic interneurons
Source: Mol Brain. 2021 Aug 24;14:130. doi: 10.1186/s13041-021-00830-6 (PMC8385910; doi:10.1186/s13041-021-00830-6)
Supplement: Supplementary file 1 — Additional file 1. Statistical table. Description of data: results of all statistical tests referenced per figure. [file 13041_2021_830_MOESM1_ESM.pdf]

Figure	Panel	Group size	Test	Statistic	P values	Statistic 2	P values 2
**1**	**e**	Veh n = 9<br>SST_{14} n = 10	2-way mixed ANOVA<br>(Condition x time)	Interaction:<br>F(2,682; 45, 600) = 5. 168	 p = 0.005		
			rmANOVA veh<br>rmANOVA SST_{14}	F(3.118 ; 24.947) = 0.255<br>F(2.247 ; 20.222) = 7.046	p = 0.864<br>p = 0.004	 Dunnett's multiple comparisons	 25-30min p = 0.025<br>30-35min p = 0.023
		Cycl + SST n = 8<br>SST_{14} n = 10	2-way mixed ANOVA<br>(Condition x time)	Interaction:<br>F(2.793; 44.692) = 10.638	 p < 0.0001		
			rmANOVA cyst+SST	F(3.151; 22.060) = 1.075	p = 0.382		
	**f**	Veh n = 9<br>SST_{14} n = 10	Univariate analysis of variance (30-35min)	F(1, 17) = 8.141	p = 0.010		
		Cycl + SST n = 8<br>SST_{14} n = 10	Univariate analysis of variance (30-35min)	F(1, 16) = 12.984	p = 0.002		
		DMSO n = 7<br>Cycl n = 8<br>Cycl + SST n = 8	2-way mixed ANOVA<br>(Condition x time)	F(8.756; 87.563) = 0.814	p = 0.0602		
			Main effect time<br>Main effect group	F(1,36; 23, 18) = 0.310<br>F(1, 17) = 0.672	p = 0.653<br>p = 0.424		
	**g**	Veh n = 9<br>SST_{14} n = 10	2-way mixed ANOVA<br>(Condition x time)<br>*Input resistance*	F(1,36; 23, 18) = 0.479	p = 0.554		
**2**	**d**	DMSO n = 12<br>Cycl. n = 10	2-way mixed ANOVA<br>(condition x time)	Interaction<br>F(3.027 ; 60.546) = 5.849	p = 0,001		
			rmANOVA Cycl<br>rmANOVA DMSO	F(3.008 ; 33.091) = 2.062<br>F(9, 63) = 7,636 p < 0.0001	p = 0.124<br>p < 0.0001	 Dunnett's multiple comparisons	 5-10min p = 0.040<br>10-15min p = 0.009<br>15-20min p = 0.006<br>20-25min p = 0.006<br>25-30min p = 0.002
	**e**	DMSO n = 12<br>Cycl. n = 10	Univariate analysis of variance (25-30min)	F(1,20) = 19.330	p = 0.0003		
**3**	**a**	Cyst. IP n = 3 mice<br>Veh n = 3 mice	Unpaired t –test	t(4) = 4.280	p = 0.049		

	**b**	Cyst. IP n = 11<br>Veh n = 11	2-way mixed ANOVA (condition x time)	F(3.491; 69.829) = 12. 118	p < 0,0001		
			rmANOVA Cyst IP	F(2.946 ; 29.458) = 7.859	p = 0,001	Dunnett's multiple comparisons	15-20min p = 0.04<br>20-25min p = 0.03<br>25-30min p = 0.005
			rmANOVA Veh	F(3.040 ; 30.401) = 6,013	p = 0,002	Dunnett's multiple comparisons	15-20min p = 0.028<br>20-25min p = 0.016<br>25-30min p = 0.0007
	**c**	Cyst. IP n = 11<br>Veh n = 11	Univariate analysis of variance (25-30min)	F(1,20) = 61.005	p < 0,0001		
	**d**	Cyst acute n = 4 mice<br>Veh n = 4 mice	Unpaired t –test	t(18) = 2.367	p = 0.029		
	**e**	Cyst acute n = 9<br>Veh = 12	2-way mixed ANOVA (condition x time)	F(3.272 ; 62.165) = 4.042	p = 0.009		
			rmANOVA Cyst acute	F(3.201; 35.215) = 2.242	p = 0,097		
			rmANOVA Veh	F(6, 48) = 2.504	p = 0,035	Dunnett's multiple comparisons	10-15min p = 0.027<br>15-20min p = 0.008<br>25-30min p = 0.027
	**f**	Cyst acute n = 9<br>Veh = 12	Univariate analysis of variance (25-30min)	F(1,19) = 22.789	p = 0.0001		
**4**	**a**	SST_{14} n = 7<br>SST_{14} + n = 8<br>DL-APV	2-way mixed ANOVA (condition x time)	F(7, 91) = 0.567	p = 0.781		
			Main effect of time	F(7, 91) = 8.214	p < 0.0001	Bonferroni's multiple comparisons	30-35min p = 0.016
			Main effect of group	F(1, 13) = 0.066	p = 0.865		
	**c**	SST_{14} n = 9<br>SST_{14} + n = 10<br>LY367385	2-way mixed ANOVA (condition x time)	F(3.191 ; 54.251) = 0.546	p = 0.664		
			Main effect of time	F(3.191 ; 54.251) = 11.313	p < 0.0001	Bonferroni's multiple comparisons	25-30min p = 0.004<br>30-35min p = 0.005
			Main effect of group	F(1,17) = 0.030	p = 0.863		
	**e**	SST_{14} n = 16	Paired t-Test	t(14) = 0.642	p = 0.531		
**5**	**c**	Veh n = 9	2-way mixed ANOVA	F(2.861; 48.637) = 1.390	p = 0.258		

		SST_{14} n = 10	(condition x time)				
			Main effect of time	F(2.861; 48.637) = 5.176	p = 0.004	Bonferroni's multiple comparisons	25-30min p = 0.029
			Main effect of group	F(1, 17) = 2.153	p = 0.161		
**6**	**d**	Veh n = 10<br>SST_{14} n = 9	2-way mixed ANOVA (condition x time)<br>*PV-INs*	F(2.557 ; 43.471) = 1.969	p = 0.141		
			Main effect of group	F(1, 17) = 0.510	p = 0.485		
			Main effect of time	F(2.557; 43.471) = 9.503	p = 0.0001	Bonferroni's multiple comparisons	10-15min p = 0.029<br>15-20min p = 0.027<br>20-25min p = 0.042<br>25-30min p = 0.021<br>30-35min p = 0.010
	**g**	Veh n = 8<br>SST_{14} n = 8	2-way mixed ANOVA (condition x time)<br>*PCs*	F(7, 98) = 0.905	p = 0.506		
			Main effect of time	F(7, 98) = 1.017	p = 0.424		
			Main effect of group	F(1, 14) = 0.945	p = 0.348		
**7**	**c**	Veh n = 11<br>SST_{14} n = 10	2-way mixed ANOVA (condition x time)	F(3.058; 58.106) = 0.829	p = 0.485		
			Main effect of time	F(3.058; 58.106) = 1.305	p = 0.281		
			Main effect of group	F(1,19) = 3.332	p = 0.084		
	**f**	DMSO n = 8<br>Cycl. n = 10	2-way mixed ANOVA (condition x time)	F(3.838; 61.402) = 1.021	p = 0.402		
			Main effect of time	F(3.838; 61.402) = 11.851	p < 0.0001	Bonferroni's multiple comparisons	20-25min p = 0.019<br>25-30min p = 0.017
			Main effect of group	F(1, 16) = 1.247	p = 0.281		
**8**	**d**	SST_{14} n = 10<br>SST_{14} + CA3 cut n = 11<br>SST_{14} + CA3 cut	**SST_{14} vs SST_{14} + CA3 cut + Gabazine**<br>2-way mixed ANOVA (condition x time)	F(2.729; 51.845) = 7.071	p = 0.001		

	+ Gabazine n = 11					
		rmANOVA SST_{14}<br>rmANOVA SST_{14}, ∅ $_{CA3}$ + Gabazine	F(2.425 ; 21.828) = 8.641<br>F(2.529 ; 25.293) = 0.974	p = 0.001<br>p = 0.498	Dunnett's multiple comparisons	0-5min p = 0.008<br>5-10min p = 0.023<br>10-15min p = 0.033<br>15-20min p = 0.033<br>20-25min p = 0.009<br>25-30min p = 0.007<br>30-35min p = 0.009
		**SST_{14} + CA3 cut vs SST_{14} + CA3 cut + Gabazine**<br>2-way mixed ANOVA (condition x time)	F(3.395; 67.906) = 6.371	p = 0.0004		
		rmANOVA SST_{14} + cut CA3	F(3.280; 32. 803) = 9.814	p < 0.0001	Dunnett's multiple comparisons	10-15min p = 0.023<br>15-20min p = 0.046<br>20-25min p = 0.027<br>25-30min p = 0.003<br>30-35min p = 0.004
**e**	SST_{14} n = 10<br>SST_{14}, ∅ $_{CA3}$ + Gabazine n = 11	Univariate analysis of variance (30-35min)	F(1, 19) = 16.828	p = 0.001		
	SST_{14}, ∅ $_{CA3}$ n = 11<br>SST_{14}, ∅ $_{CA3}$ + Gabazine n = 11	Univariate analysis of variance (30-35min)	F(1,22) = 16.133	p = 0.001		
